# Pan-Coronavirus CRISPR-CasRx Effector System Significantly Reduces Viable Titer in HCoV-OC43, HCoV-229E, and SARS-CoV-2

**DOI:** 10.1089/crispr.2022.0095

**Published:** 2023-08-14

**Authors:** Cathryn M. Mayes, Joshua L. Santarpia

**Affiliations:** ^1^ WMD Threats and Aerosol Science, Sandia National Laboratories, Albuquerque, New Mexico, USA; National Strategic Research Institute, Omaha, Nebraska, USA.; ^2^ University of Nebraska Medical Center, Omaha, Nebraska, USA; and National Strategic Research Institute, Omaha, Nebraska, USA.; ^3^ Chemical & Biological Threat Detection & Countermeasure Development, National Strategic Research Institute, Omaha, Nebraska, USA.

## Abstract

CRISPR-based technology has become widely used as an antiviral strategy, including as a broad-spectrum human coronavirus (HCoV) therapeutic. In this work, we have designed a CRISPR-CasRx effector system with guide RNAs (gRNAs) that are cross-reactive among several HCoV species. We tested the efficacy of this pan-coronavirus effector system by evaluating the reduction in viral viability associated with different CRISPR targets in HCoV-OC43, HCoV-229E, and severe acute respiratory syndrome coronavirus 2 (SARS-CoV-2). We determined that several CRISPR targets significantly reduce viral titer, despite the presence of single nucleotide polymorphisms in the gRNA when compared with a non-targeting, negative control gRNA. CRISPR targets reduced viral titer between 85% and >99% in HCoV-OC43, between 78% and >99% in HCoV-229E, and between 70% and 94% in SARS-CoV-2 when compared with an untreated virus control. These data establish a proof-of-concept for a pan-coronavirus CRISPR effector system that is capable of reducing viable virus in both Risk Group 2 and Risk Group 3 HCoV pathogens.

## Introduction

Coronaviruses have a wide host range that can lead to zoonotic spillover events, resulting in emerging human coronavirus (HCoV) diseases.^1^ At least 75% of emerging human infectious diseases have an animal origin, with over five new human diseases appearing per year.^2^ Potential spillover events of other coronaviruses from animals to humans are increasing, which may cause more pandemics in the future.^3,4^ This has led researchers to suggest developing universal coronavirus vaccines or drugs against the next coronavirus pandemic.^1,4,5^

CRISPR-based technology has become widely used as an antiviral strategy, including as a broad-spectrum HCoV therapeutic. For example, a CRISPR-Cas13 effector called PAC-MAN (prophylactic antiviral CRISPR in human cells) was developed as a viral inhibitor to degrade RNA from severe acute respiratory syndrome coronavirus 2 (SARS-CoV-2) sequences and live influenza A virus in human lung epithelial cells.^6^ Although this work represents significant advancement to the field, the efficiency and specificity of CRISPR RNAs (crRNAs) for inhibiting infection of respiratory tract cells with live SARS-CoV-2 virus still needs to be evaluated. Another study also used messenger RNA-encoded Cas13a to mitigate influenza virus A and SARS-CoV-2 infections in mice and hamsters.^7^ This demonstrates the success of CRISPR effectors in an animal model, but it is not broad-spectrum across multiple virus species.

Zeng et al. developed a proof-of-concept broad-spectrum antiviral to inhibit SARS-CoV-2 variants and other HCoV strains by designing crRNAs with an analysis pipeline that incorporates existing algorithms to compute coverage, efficiency, and specificity of crRNAs for targeting RNA viruses.^8,9^ Based on their work, the success of CRISPR-mediated pan-coronavirus inhibition is promising but still requires further investigation as the effect of the CRISPR system on many HCoV strains was evaluated with reverse transcription quantitative polymerase chain reaction (RT-qPCR), which does not directly indicate reduction in viral viability. Although RT-qPCR assays are valuable for preliminary evaluation of target efficacy, viability assays provide more insight when determining the performance of CRISPR antivirals since they directly test the presence of live virus in host cells rather than detecting nucleotide content alone.

Here, we have designed a CRISPR-CasRx effector system with guide RNAs (gRNAs) that are cross-reactive among several HCoV species. CasRx is a Cas13d type CRISPR effector derived from *Ruminococcus flavefaciens* strain XPD3002.^10^ It is an RNA-targeting endonuclease with specific cleavage that is directed by a gRNA that is composed of a 30 nucleotide (n.t.) direct repeat plus a 22 n.t. spacer that is complementary to the targeted region in the virus.

Because Cas13 effectors do not require a promotor flanking sequence in the targeted region, there is greater flexibility in which target sequence can be selected. In addition, CasRx has proven to be highly effective in mammalian cells, with >90% knockdown of RNA in mammalian cells.^10^ Using this CRISPR-CasRx system, we demonstrated significant reductions in viral viability associated with different CRISPR targets in HCoV-OC43, HCoV-229E, and SARS-CoV-2.

In addition to the highly pathogenic SARS-CoV-2, HCoV-OC43 and HCoV-229E were tested in this study to represent two of the four common HCoVs that cause mild upper respiratory tract illness and contribute to 15–30% of cases of common colds in human adults.^11^ Although these common HCoVs are not as highly pathogenic as MERS-CoV, SARS-CoV, or SARS-CoV-2, they are still a significant public health concern due to their prevalence in the community.

Further, HCoV-OC43 and HCoV-229E represent the two genera of HCoV, as HCoV-229E is an alphacoronavirus and HCoV-OC43 is a betacoronavirus. Therefore, the viruses used in this work provide a comprehensive representation of HCoV species to determine the potential use of these gRNA targets as a pan-coronavirus effector.

With the constant introduction of new variants, it is advantageous to develop viral effector systems that are resistant to the inevitable genomic mutations associated with RNA viruses by targeting conserved essential genes as we have demonstrated here.

## Materials and Methods

### Virus propagation

#### HCoV-OC43

HCoV-OC43 virus (Catalog No. VR-1558; ATCC) was propagated in HCT-8 cells (Catalog No. CCL-244; ATTC) when the host cells grew to 80–90% confluence. After aspirating media from the host cells, 2.5 mL of virus in RPMI medium was added to a T-75cm^2^ flask at a multiplicity of infection (MOI) of 0.1. Infected cells were incubated at 33°C, 5% CO_2_ for 1–2 h with continuous rocking; then, 10 mL RPMI +2% horse serum was added to the flasks, and incubation was continued for 4 days. At this time, significant cytopathic effects (CPE) were observed and included cell vacuolization and cell sloughing.

The virus was harvested by scraping the cells into the medium and quick-freezing in liquid nitrogen vapor. The viral titer was determined by performing 50% tissue culture infectious dose (TCID_50_) assay.^12^

#### HCoV-229E

HCoV-229E virus (Catalog No. VR-740; ATCC) was propagated in MRC-5 cells (Catalog No. CCL-171; ATTC) when the host cells grew to 80–90% confluence. After aspirating media from the host cells, 2.5 mL of virus in Eagle's minimum essential medium (EMEM) was added to a T-75cm^2^ flask at an MOI of 0.1. Infected cells were incubated at 35°C, 5% CO_2_ for 1–2 h; then, 10 mL EMEM +2% fetal bovine serum was added to the flasks, and incubation was continued for 6 days. At this time, significant CPE were observed and included cell rounding and cell sloughing.

The virus was harvested by scraping the cells into the medium and quick-freezing in liquid nitrogen vapor. The viral titer was determined by performing a TCID_50_ assay.

#### SARS-CoV-2

The CDC reference strain SARS-CoV-2 USA-WA1/2020 was used in this work. The strain was deposited by the Centers for Disease Control and Prevention and obtained through BEI Resources, NIAID, NIH: SARS-Related Coronavirus 2, Isolate USA-WA1/2020, NR-52281. SARS-CoV-2 virus was propagated in Vero E6 cells when the host cells grew to ∼90% confluence. After aspirating media from the host cells, 500 mL of virus in DMEM +5% fetal bovine serum was added to a Corning HYPERFlask at an MOI of 0.01–0.1. Infected cells were incubated at 35°C, 5% CO_2_ for 72 h. Once substantial CPE was achieved throughout the infected HYPERFlask, the media was harvested from the HYPERFlask into 50 mL conical tubes.

All tubes were centrifuged at 2000 *g* for 10 min at 4°C, and the supernatant was collected and pooled into a single bottle. Virus was concentrated using a VivaFlow with a peristaltic pump recirculating at 200–400 mL/min. Concentrated virus was aliquoted and stored at −80°C, and the stock was titered via plaque assay.

#### CRISPR target design and selection

CRISPR targets were designed with single nucleotide polymorphisms (SNPs), as previously described.^13^ Briefly, NCBI BLAST analyses and sequencing alignment were used to identify regions within the RNA-dependent RNA polymerase (RdRp) and nucleocapsid (N) genes that had the highest percent identity between HCoV-OC43 and SARS-CoV-2. Additional gRNAs were then designed that had between 1 and 3 SNPs. These gRNA sequences were cloned into the plasmid pXR003 (Addgene plasmid #109053) for delivery into host cells, as previously described.^13^ Of the 17 total gRNAs previously designed and tested, we selected the gRNAs that had the highest activity in HCoV-OC43 as determined by RT-qPCR (Table 1).

**Table 1.** Guide RNA sequences and human coronavirus genome target locations

gRNA name	gRNA sequence (5′–3′)	Target location in HCoV-OC43	Target location in HCoV-229E	Target location in SARS-CoV-2
RdRp_ctrl	UUAUGGGUUGGGAUUAUCCUAA	15,181–15,202	14,311–14,332 with 3 SNPs	15,281–15,302
RdRp	UGGACCUCAUGAAUUUUGUUCA	15,761–15,782	14,892–14,911 with 2 SNPs	15,862–15,881 with 3 SNPs
RdRp _A	AGGACCUCAUGAAUUUUGUUCA	15,761–15,782 with 1 SNP	14,892–14,911 with 1 SNP	15,862–15,881 with 2 SNPs
RdRp _ABC	AGGACCUCAUGAAUUUUGCUCU	15,761–15,782 with 3 SNPs	14,892–14,911 with 1 SNP	15,862–15,881
N	CACGAUGGUAUUUUUACUAUCU	29,513–29,534	25,903–25,923 with 5 SNPs	28,590–28,611 with 3 SNPs
N_B	CACGAUGGUAUUUCUACUAUCU	29,513–29,534 with 1 SNP	25,903–25,923 with 4 SNPs	28,590–28,611 with 2 SNPs
N_ABC	CAAGAUGGUAUUUCUACUACCU	29,513–29,534 with 3 SNPs	25,903–25,923 with 3 SNPs	28,590–28,611
Neg gRNA	AUCUAUUGUUCCGACGUAUUAU	N/A	N/A	N/A

gRNA, guide RNAs; HCoV, human coronavirus; RdRp, RNA-dependent RNA polymerase; SARS-CoV-2, severe acute respiratory syndrome coronavirus 2.

#### Transfection of Vero cells

Vero cells were transfected simultaneously with a plasmid encoding CasRx (pXR001: EF1a-CasRx-2A-EGFP was a gift from Patrick Hsu [Addgene plasmid #109049; http://n2t.net/addgene:109049; RRID:Addgene_109049]) and a plasmid encoding a gRNA (pXR003: CasRx gRNA cloning backbone was a gift from Patrick Hsu [Addgene plasmid #109053; http://n2t.net/addgene:109053; RRID:Addgene_109053]).^10^ Vero cells were seeded on a 24-well plate containing 5 × 10^4^ cells/well and incubated overnight until 70–90% confluent.

Cells were transfected with 500 ng DNA of each plasmid using a calcium phosphate transfection kit (Catalog No. L3000015; Invitrogen) according to the manufacturer's protocol, adjusting the volumes for the smaller volumes of the 24-well plate.^14^ Cells were incubated at 37°C, 5% CO_2_ overnight. The media were changed, and incubation continued at 37°C for 24 h. Although the plasmids encode a bleomycin resistance selection marker, no selection was employed post-transfection to reflect the cellular conditions of an *in vivo* model more accurately where antibiotic selection of a plasmid is impractical.^15^

Transfection efficiency of CRISPR plasmids in Vero cells was previously determined using microscopy to evaluate the percent of cells expressing green fluorescent protein (GFP). This was accomplished by comparing the manual counts of GFP-expressing cells with non-GFP-expressing cells 48 h post-transfection. We found that the average percentage of Vero cells expressing GFP was 60.7% with a standard error of ±1.70%.^13^

#### Infecting transfected Vero cells with HCoV

After 48 h post-transfection with CRISPR plasmids, Vero cells were infected with virus at an MOI of 0.01. Infected Vero cells were incubated for 1.5 h with continuous rocking. Growth media were aspirated from the cells and replaced with fresh growth media. Incubation continued for 4 days, and each day post-infection 200 μL supernatant containing viral lysate was collected to determine viral titer with a TCID_50_ assay.

#### TCID_50_ assay

Vero cells were seeded on a 96-well plate with each well containing 10,000 cells in 100 μL DMEM-2 media and incubated at 37°C and 5% CO_2_ for 24 h. Viral lysates were serially diluted using 10-fold dilutions; then, 100 μL of each virus dilution was added to the 96-well plate in triplicate. Samples were incubated at 37°C and 5% CO_2_ for 4–7 days until CPE developed. Viable viral titer was determined using the Spearman–Kärber statistical method.

This method uses the following equation to calculate titer: 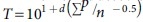, where *T* = viral titer in TCID_50_/mL; *d* = log_10_ of the dilution factor; *p* = number of tests with positive CPE; and *n* = total number of tests per dilution.

### Statistical analysis

Statistical significance was calculated using one-sided *t*-tests to determine the differences between the experimental gRNA and the control groups. Data were log-transformed before performing the analyses to convert the lognormal distribution into a Gaussian distribution required for the *t*-test. Statistical significance was defined as a *p*-value ≤0.05. All statistical analyses were performed in GraphPad Prism software (v 9.4.0; GraphPad Software, LLC).

## Results

### Design and downselection of gRNAs

We previously designed CRISPR targets that are potentially cross-reactive among different HCoV species by targeting highly conserved regions of essential genes, including the RdRp gene and N gene.^13^ The gRNAs containing SNPs were also designed to determine CasRx efficacy when gRNAs are not perfectly complimentary to their target sequences. We previously determined the performance of each gRNA in HCoV-OC43 via RT-qPCR by evaluating the reduction in viral RNA compared with a control, non-targeting gRNA (Neg gRNA).^13^ Based on these data, we downselected the gRNAs with the highest efficacy to test for reduction in viability across multiple HCoVs, including HCoV-OC43, HCoV-229E, and SARS-CoV-2.

**FIG. 1.**
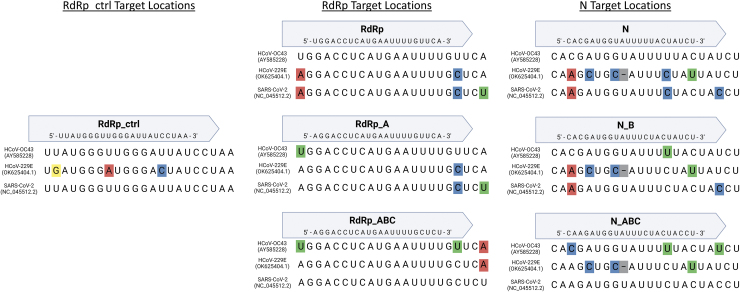
Comparison of CRISPR target locations in HCoV-OC43, HCoV-229E, and SARS-CoV-2 genomes. The SNPs in each genome are highlighted based on nucleotide. SARS-CoV-2, severe acute respiratory syndrome coronavirus 2; SNPs, single nucleotide polymorphisms;.

We specifically tested N and RdRp guides with no mismatches compared with HCoV-OC43 (N, RdRp, and RdRp_ctrl), guides with no mismatches compared with SARS-CoV-2 (N_ABC, RdRp_ABC, and RdRp_ctrl), and guides with one SNP in the gRNA compared with HCoV-OC43 (N_B and RdRp_A). In addition, we tested a non-targeting negative control gRNA (Neg gRNA). The sequences and targeting locations in each virus are summarized in Table 1 and Figure 1.

In addition to the three HCoVs tested in this study, we evaluated other SARS-CoV-2 variants for target conservation and found that the targeting sequences are identical in the Alpha, Beta, Delta, and Omicron variants. Variant sequence analyses were performed using the following GenBank sequences: OP879258, OP880662, MZ437368, and ON026021, respectfully.

We further evaluated the conservation of gRNA sequences among the remaining HCoV species *in silico* to determine the potential use of these targets as a pan-HCoV effector. This analysis indicated high sequence conservation among all HCoV species and demonstrates the potential for these targets to be used as a pan-coronavirus effector (Table 2).

**Table 2.** Conservation of guide RNA sequences in remaining human coronavirus

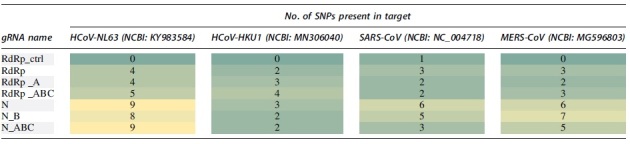

HCoV-NL63 targeting regions include 14,177–14,196 for RdRp_ctrl, 14,805–14,826 for RdRp, and 22,707–22,728 for N. Targeting regions in HCoV-HKU1 include 15,304–15,325 for RdRp_ctrl, 15,990–16,011 for RdRp, and 28,684–28,705 for N. SARS-CoV targeting regions are 15,211–15,232 for RdRp_ctrl, 15,791–15,812 for RdRp, and 28,439–28,460 for N. In MERS-CoV, targeting regions include 15,178–15,199 for RdRp_ctrl, 15,758–15,779 for RdRp, and 28,734–28,755 for N.

### CRISPR plasmids transfection and infection with HCoV

To test the efficacy of gRNAs to reduce viral viability, CRISPR plasmids were transfected into Vero cells, and each individual gRNA was tested against HCoV-OC43, HCoV-229E, and SARS-CoV-2 separately as previously described.^13^ To accomplish this, a plasmid encoding CasRx was simultaneously transfected with a plasmid encoding an individual gRNA into Vero cells using the calcium phosphate transfection method.^14,16^

Host cells were then infected with virus at an MOI of 0.01 after 48 h post-transfection. After infection, supernatant containing lysed virus was collected, and virus viability assays were performed on each sample. Samples containing HCoV-OC43 or HCoV-229E were collected for analysis 2 days post-infection, and SARS-CoV-2 samples were analyzed 4 days post-infection.

### CPE in HCoVs

**FIG. 2.**
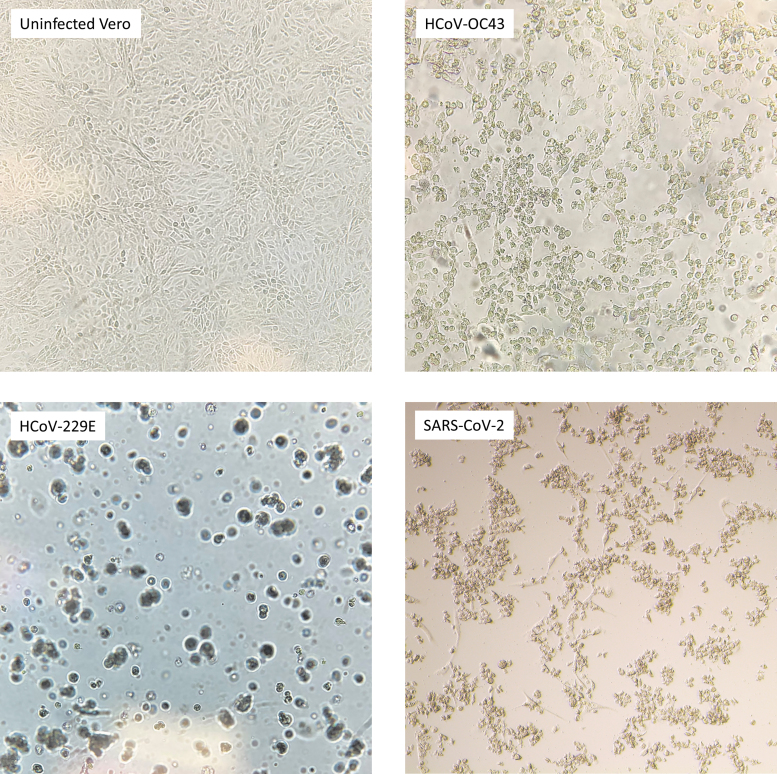
CPE caused by each HCoV in Vero cells. Uninfected Vero cells are shown as a negative control for comparison. CPE, cytopathic effect; HCoV, human coronavirus.

We performed TCID_50_ assays using Vero cell monolayers on 96-well plates to determine how each gRNA affects viability in all HCoV tested. We visualized CPE with an inverted microscope as an endpoint for TCID_50_ analysis. CPE for HCoV-OC43 included cell vacuolization, rounding, and sloughing. In cells infected with HCoV-229E and SARS-CoV-2, CPE included cell rounding and sloughing (Fig. 2).

### CRISPR targets significantly reduce viral titer despite presence of SNPs in gRNA

**FIG. 3.**
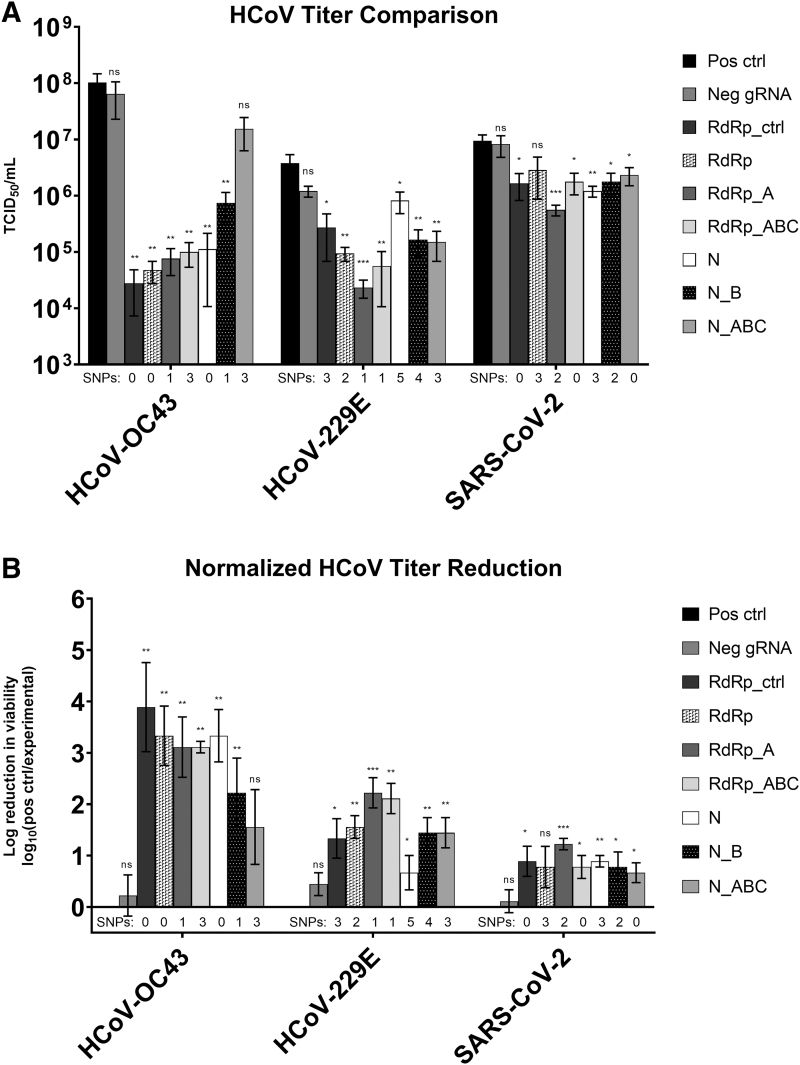
HCoV TCID_50_ results following transfection with CasRx and individual gRNAs. Each sample was tested in triplicate, and error bars represent standard error. **(A)** Raw titer of each sample in TCID_50_/mL. **(B)** Log reduction in viability compared with the positive control sample. TCID_50_, 50% tissue culture infectious dose. Statistical significance was calculated using one-sided t-tests to determine the differences between the experimental gRNA and the positive control groups. ns, *p* < 0.05; *, *p* ≤ 0.05; **, *p* ≤ 0.01; ***, *p* ≤ 0.001.

The efficacy of each gRNA was tested against each HCoV species in triplicate with three independent biological replicates to determine statistical significance of the data (Fig. 3). We performed one-tailed *t*-tests to compare reduction in viral concentration associated with each gRNA CRISPR target to either the untreated positive control virus or the non-targeting Neg gRNA (Table 3).

**Table 3.** One-tailed *t*-test comparing reduction in viral concentration associated with each guide RNA CRISPR target

CRISPR target	HCoV-OC43		HCoV-229E		SARS-CoV-2	
	Comparison with Pos Ctrl	Comparison with Neg gRNA	Comparison with Pos Ctrl	Comparison with Neg gRNA	Comparison with Pos Ctrl	Comparison with Neg gRNA
RdRp_ctrl	0.0023	0.0025	0.0128	0.0323	0.0237	0.0457
RdRp	0.0011	0.0011	0.0011	0.0011	0.0621 (ns)	0.0982 (ns)
RdRp_A	0.0011	0.0011	0.0008	0.0010	0.0007	0.0038
RdRp_ABC	0.0025	0.0028	0.0045	0.0080	0.0125	0.0353
N	0.0027	0.0031	0.0353	0.1870 (ns)	0.0024	0.0125
N_B	0.0065	0.0079	0.0074	0.0167	0.0125	0.0353
N_ABC	0.1170 (ns)	0.1460 (ns)	0.0040	0.0079	0.0275	0.0659 (ns)
Neg gRNA	0.3214 (ns)	—	0.0581 (ns)	—	0.3219 (ns)	—

ns indicates the percent reduction is not significantly different from that of the HCoV-OC43 control or negative gRNA. Statistical significance was calculated using one-sided *t*-tests to determine the differences between the experimental gRNA and the control groups. Data were log-transformed before performing the analyses to convert the lognormal distribution into a Gaussian distribution required for the *t*-test. Statistical significance was defined as a *p*-value ≤0.05.

ns, not significant.

Comparing the values with the Neg gRNA accounts for any reduction in viability associated with transfection methods alone, whereas the virus positive control confirms that the CRISPR targets significantly reduce viral titer. The resulting viral titer after exposure to the Neg gRNA was not significantly different from the titer of the virus positive controls (*p* = 0.3214 for HCoV-OC43; *p* = 0.0581 for HCoV-229E; *p* = 0.3219 for SARS-CoV-2), indicating that the transfection method alone does not significantly affect viability.

### HCoV-OC43

When CRISPR targets were tested against HCoV-OC43, viral titer decreased by ≥85% for all CRISPR gRNAs tested compared with the positive control (Table 4). All gRNAs were significantly different than the Neg gRNA except N_ABC, which had a *p*-value of 0.1460. RdRp_ctrl had the greatest effect on HCoV-OC43 and reduced viral titer by 99.97% (*p* = 0.0025).

**Table 4.** Percent reduction of human coronavirus titer compared with virus only control following transfection with CRISPR components

CRISPR target	% Reduction in HCoV-OC43 compared to positive control (%)	% Reduction in HCoV-229E compared to positive control (%)	% Reduction in SARS-CoV-2 compared to positive control (%)
RdRp_ctrl	99.97	92.86	82.54
RdRp	99.95	97.52	69.65
RdRp_A	99.93	99.39	94.07
RdRp_ABC	99.90	98.53	81.25
N	99.89	78.44	87.21
N_B	99.28	95.68	81.25
N_ABC	85.00	96.05	75.28
Neg gRNA	37.38	68.35	12.89

### HCoV-229E

When HCoV-229E was treated with CRISPR targets, viable viral titer decreased between 78.44% and 99.39% compared with the untreated HCoV-229E positive control (Table 4). All targets were statistically different than the positive control, and all gRNAs except N (*p* = 0.1870) were statistically different than the Neg gRNA.

### SARS-CoV-2

All CRISPR targets significantly reduced SARS-CoV-2 titer compared with the Neg gRNA except RdRp (*p* = 0.0982) and N_ABC (*p* = 0.0659). The reduction in SARS-CoV-2 viable titer ranged from 69.65% to 94.07% (Table 4).

## Discussion

The pan-coronavirus CRISPR effectors developed and evaluated in this work are highly effective against all three HCoVs tested. CRISPR targets reduced viral titer between 85% and >99% in HCoV-OC43, between 78% and >99% in HCoV-229E, and between 70% and 94% in SARS-CoV-2. Among all gRNAs tested, RdRp_A had the greatest efficacy among all three viruses, with >99% reduction in HCoV-OC43 and HCoV-229E, and 94.07% reduction in SARS-CoV-2.

These data indicate that RdRp_A is a promising target to utilize as a pan-coronavirus CRISPR effector. RdRp_ctrl is also a promising candidate for an individual pan-coronavirus CRISPR target as it has high efficacy among the HCoVs tested in the present study (Table 4) and is highly conserved among all HCoV species (Table 2).

Although previous work has evaluated how CRISPR targets can significantly reduce HCoV RNA using RT-qPCR analysis,^8,13^ it is important to also determine the effect that CRISPR targets have on viable viral titer as we have demonstrated here. RT-qPCR results only provide information on RNA integrity and do not necessarily indicate the presence of viable virus^17^; therefore, viability assays provide more insight when determining the performance of CRISPR antivirals, especially in the context of therapeutic development. In this study, we have shown that our gRNA targets have a greater effect on viral viability than what was found using RT-qPCR assays.

For example, when testing these CRISPR targets with RT-qPCR assays, the highest percent reduction in viral RNA was 91%^13^; however, when testing CRISPR targets in HCoV-OC43 using viability assays, there was up to a 4-log reduction in infectious virus.

Our previous work with these pan-coronavirus CRISPR targets suggested that CasRx is tolerant to SNPs in the gRNA sequence based on preliminary RT-qPCR data that showed reduction in viral RNA,^13^ and the results here support that observation as well. We have demonstrated that CRISPR targets can effectively reduce viral titer, even when SNPs are present in HCoV-OC43, HCoV-229E, and SARS-CoV-2 (Tables 1 and 3), which is an important finding regarding the safe design of CRISPR targets when using the CasRx effector.

We found that the efficacy of each gRNA tested directly corresponded to the number of SNPs present in both HCoV-OC43 and HCoV-229E. As the number of SNPs increased, the resulting reduction in viral titer decreased in both of these viruses; however, the CRISPR targets still significantly reduced viral titer despite the presence of SNPs in many of the gRNAs tested (Table 3). gRNA SNPs seemed to have less impact on the reduction of SARS-CoV-2 titer.

For example, N contains three SNPs compared with the SARS-CoV-2 genome; however, it reduced the viral titer more than N_ABC, which contained no SNPs. These findings verify that CasRx is tolerant of mismatches in three different HCoVs. Further, by targeting conserved regions of essential genes, this antiviral method is more resistant to viral escape mutations,^8^ as demonstrated by the complete conservation of our CRISPR targets in the Alpha, Beta, Delta, and Omicron variants of SARS-CoV-2. This is critical for ssRNA viruses such as HCoVs, which rapidly mutate as new variants arise.^18,19^

Although we show significant viral reduction with individual CRISPR targets, the effects may become even more pronounced when multiple targets are coupled in a multiplexed cocktail approach.^20^ Using this approach, multiple guides may be used to target one gene, or multiple genes could be targeted simultaneously. It has been previously demonstrated that targeting multiple gRNAs to a single genetic locus enhances DNA editing efficiency,^21,22^ and optimized efficiency was achieved when clusters of 3–4 gRNAs were used to target a single gene.^23^

In addition to increasing the overall antiviral activity, multiplexing CRISPR targets can also prevent viral resistance to CRISPR therapy.^24–26^ Combinations of at least three different gRNAs are typically involved to counter viral escape mutants.^27,28^

Because this pan-coronavirus CRISPR effector system is cross-reactive among multiple HCoV species, the efficacy should also be tested when simultaneously coinfecting cells with more than one HCoV. The current work tests individual gRNAs in each HCoV separately; however, this tool would be more advantageous if it can effectively target two or more viruses simultaneously.

### Potential applications

There are broad applications where this pan-coronavirus CRISPR effector system can be utilized. First, this tool may be developed further to be used as an antiviral therapeutic.^29^ CRISPR-based therapeutics have shown promising success, even resulting in a Phase I/II clinical trial for a CRISPR-based antiviral targeting HIV.^30^ CRISPR-Cas13-based antiviral therapy may be more sensitive and specific than traditional treatments since it focuses on cleaving viral RNA inside infected cells opposed to traditional antiviral therapies that often trigger the human immune system to recognize viral proteins and diminish entry.^31^ To develop this CRISPR effector system into a therapeutic, further work will need to be conducted involving an appropriate delivery mechanism for the CRISPR reagents to the patient and determining dosage and treatment regimens.^8^

Another potential application for the gRNA targets presented here involves diagnostics and detection. There are several methods to utilize CRISPR targets as a detection and diagnostic tool,^32–34^ including fluorescence-based detection,^35,36^ lateral flow detection,^37^ or electrochemical microfluidics.^38^ These methods detect the presence of viruses by providing a visual or fluorescence signal if CRISPR cleavage has occurred at the targeted location.

In addition, these techniques can detect viruses with a limit of detection in the attomolar range, making them an extremely sensitive, specific, and fast detection technology.^39^ The work here further advances the CRISPR biodetection application space by extending this detection tool from species-level detection to broad-spectrum detection at the family level.

Biodetection technologies directed at the viral family level offer the potential to detect emerging pathogens that otherwise would evade traditional, agent-specific detection methods such as qPCR. Further, rapidly detecting an unknown pathogen at the family level can provide immediate information on which countermeasures may be effective at the point of need without prior characterization of the pathogen.

## Conclusions

Ultimately, we have established a highly effective pan-coronavirus CRISPR system that is capable of reducing viable virus in both Risk Group 2 and Risk Group 3 HCoV pathogens and has the potential to be used in a broad application space.
